# Gaseous flow through heterogeneous, partially connected networks of pipes

**DOI:** 10.1038/s41598-018-33374-2

**Published:** 2018-10-08

**Authors:** Yves Bernabé

**Affiliations:** Earth, Atmospheric and Planetary Sciences Department, Massachusetts Institute of Technology, Cambridge, Massachusetts USA

## Abstract

Simulations of flow of an ideal gas through heterogeneous simple cubic pipe networks with different pipe radius distributions and variable bond coordination numbers were performed. Networks with monomodal and bimodal radius distributions were constructed. A very wide range of Knudsen numbers was achieved. Flow simulations of purely viscous gases and incompressible liquids were also carried out for comparison. The permeability to gas in the purely viscous regime was larger than the permeability to an incompressible liquid. Based on a variety of computational tests, this result was likely not a numerical artifact. The simulated macroscopic flow behavior differed from the underlying single pipe model, depending on the radius distribution, network connectivity and magnitude of the externally applied pressure gradient, and was compatible with the Klinkenberg analysis only when the maximum Knudsen number used in each simulation was lower than 1. In this condition, the Klinkenberg coefficient was nearly proportional to the inverse of the network hydraulic radius while the effect of the radius distribution was weak and that of the network connectivity essentially negligible. The bimodal simulations displayed a typical percolation behavior, with the Klinkenberg coefficient remaining constant as long as the large pipe population was connected.

## Introduction

Gas flow through pipes and ducts has been a problem of great interest to physicists for nearly 150 years^1^. It was early recognized that, at high gas pressures, gas flow obeys identical laws as liquid flow, although the compressibility of gas is much higher than that of liquids. But gas flow appeared utterly different at low gas pressures. Careful experiments showed that, with all other variables being held constant, the apparent transmissivity of long pipes increased with decreasing mean gas pressure. Moreover, the magnitude of this effect was observed to increase when thinner pipes were used.

Analysis of the experimental evidence demonstrated that gas flow depends on the dimensionless Knudsen number *K*_n_ = λ/*R*, where *R* is the pipe radius and λ = (μ/*p*) (π*R*_*g*_*T*/2)^1/2^ the mean free path of the gas molecules, with *p* denoting the gas pressure, μ the viscosity, *R*_*g*_ the specific gas constant and *T* the temperature. Two extreme flow regimes can be described depending on *K*_n_. At very high gas pressures or, equivalently, very low *K*_n_, λ is infinitesimally small and the gas motion is controlled by the collisions of gas molecules among themselves. In this limit, the gas can be described as a viscous fluid subject to the usual equations of continuum fluid dynamics, supplemented with a no-slip boundary condition along the internal pipe wall. For long pipes, the mass flow rate is derived from the compressible Poiseuille flow formula:

1 $${\dot{m}}_{v}=-\,\frac{{\rm{\pi }}{R}^{4}\bar{p}}{8\,{\rm{\mu }}Z{R}_{g}T}\frac{{\rm{\Delta }}p}{L},$$

where *L* is the pipe length, *Z* the gas compressibility factor (*Z* = 1 for ideal gases), $\bar{p}$ the mean gas pressure and Δ*p* the pressure difference driving the flow.

At very low gas pressures (i.e., very large *K*_n_), λ is many times greater than the pipe radius, intermolecular collisions are essentially inexistent and the gas motion is controlled by the collisions of gas molecules with the internal pipe wall. In these conditions, continuum fluid dynamics and the concept of viscosity become inapplicable. The proper description is instead provided by Boltzmann’s equation, which can be shown to reduce to a diffusion equation where the independent variable is gas pressure^2^. This flow regime is usually called free molecular or Knudsen diffusion. In long pipes, the Knudsen diffusion mass flow rate is expressed as^3^:

2 $${\dot{m}}_{K}=-\,\frac{4}{3Z}{R}^{3}\frac{{\rm{\Delta }}p}{L}\sqrt{\frac{2{\rm{\pi }}}{{R}_{g}T}}.$$

One implication of equation 2 is that, for ideal gases, Knudsen diffusion is insensitive to the mean gas pressure.

The limits of the viscous flow regime and Knudsen diffusion are usually estimated to be *K*_n_ < 0.001 and *K*_n_ > 10, respectively. The problem is to discover what happens in the transition range, 0.001 < *K*_n_ < 10. It was early realized that, for *K*_n_ slightly greater than 0.001, the concept of viscosity and the Navier-Stokes equation can be retained. The net effect of the increasing number of molecule-wall collisions is merely to change the gas velocity boundary condition along the internal pipe wall (see Klinkenberg^4^ and references therein). The finite tangential gas velocity developing along the internal pipe wall can be expressed based on the kinetic theory of gases as:

3 $${u|}_{r=R}=-\,\frac{2-{\rm{\sigma }}}{{\rm{\sigma }}}{\rm{\lambda }}{\frac{\partial u}{\partial r}|}_{r=R},$$

where σ, the tangential momentum accommodation coefficient (TMAC), is the fraction of wall-colliding molecules that undergo diffusive (as opposed to specular) reflections. The TMAC is usually assumed equal to 1, although lower values have been inferred from experimental data and numerical simulations^5–9^. By application of a perturbation method and equation 3 to long cylindrical pipes^10^, the mass flow rate is found to be equal to:

4 $${\dot{m}}_{S}=-\,\frac{{\rm{\pi }}{R}^{4}\bar{p}}{8\,{\rm{\mu }}Z{R}_{g}T}\frac{{\rm{\Delta }}p}{L}(1+4\frac{2-{\rm{\sigma }}}{{\rm{\sigma }}}{\bar{K}}_{{\rm{n}}}),$$

where ${\bar{K}}_{{\rm{n}}}$ is the Knudsen number evaluated at the mean gas pressure (for simplicity, the mean Knudsen number will be denoted *K*_n_ hereafter). Equation 4 describes the so-called slip flow regime and is normally deemed to hold only up to *K*_n_ ≈ 0.1, although the Klinkenberg analysis, which is based on the slip flow concept, is often used for much higher values of *K*_n_.

How to model the transitional gas flow regime, i.e., for 0.1 < *K*_n_ < 10, is not totally settled at present. According to one school of thought, ${\dot{m}}_{K}$ is negligible in comparison to ${\dot{m}}_{V}$ when *K*_n_ approaches zero while the reverse is true for very high values of *K*_n_. Thus, the sum ${\dot{m}}_{V}+{\dot{m}}_{K}$ approaches the correct limits at high and low Knudsen numbers and can be assumed to provide an estimate of the mass flow rate with relatively small errors for any value of *K*_n_ in the transitional flow regime^11^. This notion is theoretically justified if viscous flow and molecular diffusion can be considered to be independent and uncoupled^12^. A number of variations of this model were used to study gas flow in porous media^3,13–17^.

An alternative idea for modeling transitional flow is to note that the right-hand side of equation 3 can be viewed as the first term of a Taylor expansion in λ or, equivalently, *K*_n_. The idea is then to model transitional flow by adding higher-order terms^18^. A variety of second-order slip models have been proposed in recent years^1,8,9,19,20^. Among them, the Beskok and Karniadakis’s model^7^ (BK) is presently receiving the most attention^21–25^. According to the BK model, the mass flow rate is given by:

5 $${\dot{m}}_{T}=-\,\frac{{\rm{\pi }}{R}^{4}\bar{p}}{8\,{\rm{\mu }}Z{R}_{g}T}\frac{{\rm{\Delta }}p}{L}F({K}_{{\rm{n}}}),$$

with

6 $$F({K}_{{\rm{n}}})=(1+\frac{2{{\rm{\alpha }}}_{0}}{\pi }{K}_{{\rm{n}}}\mathrm{ArcTan}({{\rm{\alpha }}}_{1}{K}_{{\rm{n}}}^{{{\rm{\alpha }}}_{2}}))(1+\frac{2-{\rm{\sigma }}}{{\rm{\sigma }}}\frac{4{K}_{{\rm{n}}}}{1-{b}_{BK}{K}_{{\rm{n}}}}).$$

In the BK original work, the ideal gas law and total momentum accommodation were assumed, implying *Z* = 1 and σ = 1^7^. The function *F* defined by equation 6 was then empirically determined by comparison with experimental and numerical simulation data^7^. The empirical values *b*_*BK*_ = −1, α_0_ = 6/5, α_1_ = 4 and α_2_ = 2/5 were inferred from the Loyalka and Hamoodi’s computational data^26^. With these values, the function *F* can be approximated by

$$F=1+4{K}_{{\rm{n}}}+48/5{\rm{\pi }}{{K}_{{\rm{n}}}}^{7/5}+O({{K}_{{\rm{n}}}}^{8/5}),$$

when *K*_n_ approaches zero, while it becomes asymptotically linear with a slope equal to 6, when *K*_n_ grows to infinity. Although the value of α_0_ mentioned above provides the best fit to the Loyalka and Hamoodi’s data^26^, it is not consistent with the Knudsen diffusion limit. Civan^22^ proposed to use α_0_ = 64/15π instead (he also modified the first factor in the right-hand side of equation 6 to avoid the ArcTan function). This change of α_0_ only causes minor changes to *F* in the low Knudsen number limit (i.e., the leading terms are unchanged) and produces a steeper slope (32/3) in the large Knudsen number limit. The BK input values (including *Z* = 1 and σ = 1) will be used hereafter.

**Figure 1:**
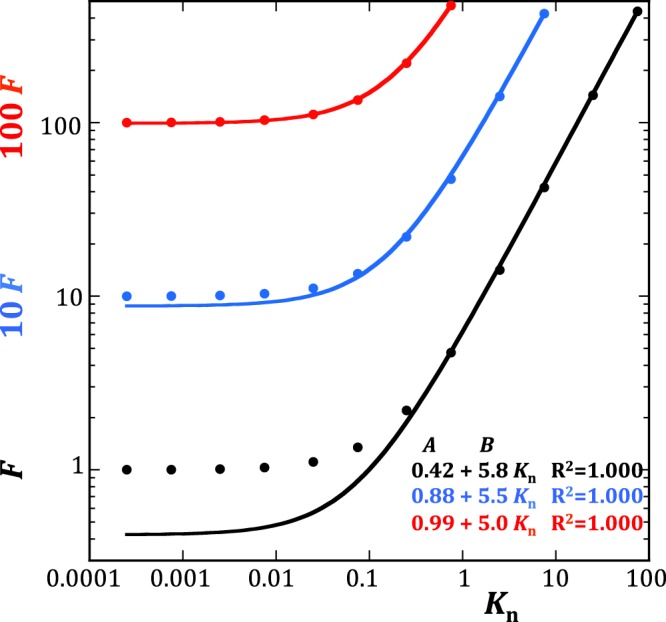
Three discrete representations of the function *F* spanning intervals of Knudsen numbers reaching a maximum value *K*_max_ equal to 74 (black dots), 7.4 (blue dots) and 0.74 (red dots). The diagram shows the function *F* multiplied by 10 (blue) and 100 (red) for better visibility. The straight lines best fitting these discrete sets of data-points are indicated as solid curves (the curvature is a result of the log-log scale) in matching colors. The intercept *A* is clearly lower than 1, increased with decreasing *K*_max_ and asymptotically reached 1 when *K*_max_ dropped below 1.

According to Klinkenberg^4^, the ratio of the gas permeability to the intrinsic permeability of porous media, *k*_*g*_/*k*, is a linear function of 1/*p* of the form, *k*_*g*_/*k* = 1 + *b*_K_/*p*, where the Klinkenberg coefficient *b*_K_ is a positive constant. Since $F={\dot{m}}_{T}/{\dot{m}}_{V}$ for a single pipe is analogous to *k*_*g*_/*k*, it is interesting to assess how different *F* is from the Klinkenberg linear model, which, in the case of *F*, can be rewritten as *F* = *A* + *B K*_n_, where *A* should be equal to unity. I constructed discrete representations of *F* spanning intervals of Knudsen numbers reaching higher and higher maximum values *K*_max_ as shown in Fig. 1 (I used the log-log scale for a better visibility of the low *K*_n_ region). Straight lines did fit these discrete sets of data-points very well in all cases (the goodness-of-fit R^2^ coefficients were all equal to 0.9999 or better), but, as can be expected from the non-linearity of equation 6, *A* and *B* varied with *K*_max_. The intercept *A* was always lower than 1, increasing with decreasing *K*_max_ and asymptotically reaching 1 only when *K*_max_ dropped below 1. The slope *B* decreased with *K*_max_, presumably approaching 4, the value predicted by equation 4, for vanishing values of *K*_max_ (Fig. 1). These observations suggest that standard linear regression analysis applied to limited discrete datasets cannot easily detect the slight upward curvature of *F* even for *K*_n_ as high as 100. The implication is that the Klinkenberg linear extrapolation of experimental gas permeabilities to zero inverse gas pressure 1/*p* may lead to significant underestimation of the intrinsic permeability *k* in porous media with a behavior analogous to that expressed by equation 6. The obvious solution to this problem is, of course, to measure the intrinsic permeability directly by performing gas flow tests at very high gas pressures, i.e., in conditions where equation 1 applies. But this method cannot work in practice owing to the deformability of real porous media and the ensuing pressure sensitivity of intrinsic permeability.

One problem of major interest is to extend the single pipe models discussed above to general porous media. Among many possible approaches, one of the most popular is to follow the equivalent channel model (ECM) method^27,28^. According to ECM, the complex features of the pore space can be lumped into a few parameters characterizing a single representative pore channel (e.g., volume fraction, cross-sectional size and tortuosity factor). If the equivalent channel is idealized as a simple cylindrical pipe, it is easy to demonstrate that the intrinsic permeability is given by^27,28^:

7 $$k=\frac{{\rm{\varphi }}{R}_{H}^{2}}{8{\rm{\tau }}},$$

where ϕ denotes the porosity, τ the tortuosity (i.e., ratio of the pipe length to its projection in the nominal flow direction) and the characteristic pipe radius is defined as the hydraulic radius *R*_*H*_ (i.e., twice the volume-to-surface ratio of the pore space). Note that ϕ and *R*_*H*_ can be physically measured in most porous media, while τ is not directly accessible. Variations of the ECM approach have been frequently used to model gas flow, sometimes in combination with numerical simulations of gas flow through three-dimensional reconstructions of the pore space^1,14,20,22–24,29^. The main drawback of the ECM approach is that it does not account for the very large variability in pore shape and size typical of most porous media. One simple and versatile alternative approach is to simulate gas flow through heterogeneous networks of pipes by incorporating the single pipe models discussed above in the Kirchoff equations^3,15–17^. Imperfectly connected networks can also be constructed, allowing investigation of the effect of pore connectivity on gas flow^17^.

## Numerical Procedures

The aim of this work is not to verify/falsify the single pipe models mentioned above, but to investigate their extension to heterogeneous and partially connected porous media. The network simulation approach is most certainly indicated for this purpose. Since the BK model has received greater support from experimental and numerical simulation data than other contending models, I elected to use it (i.e., equations 5 and 6) in my network simulations. The numerical procedures employed here are based on the Bernabé *et al*.’s^30^ workflow template for studying liquid flow and ionic conduction through heterogeneous networks. The numerical procedures followed in the present study are briefly sketched below (more details about the Bernabé *et al*.^30^ procedures can also be found in previous related papers^17,31^).

### Construction of network realizations

Two kinds of network realizations were constructed, corresponding to monomodal and bimodal radii distributions. In monomodal realizations, the radii *R*_*i*_ of individual pipes were randomly assigned according to log-uniform distributions such that the hydraulic radius of the network *R*_*H*_ honored pre-selected values (note that the mean pipe radius is not equal to the hydraulic radius; see Bernabé *et al*.^30^ for details). Since the Knudsen number depends on pore size, I considered five values of *R*_*H*_, namely 30, 10, 3, 1 and 0.3 10^−6^ m (the pipe length was *L* = 10 *R*_*H*_). The coefficient of variation of the distribution, *CV*, is a convenient measure of the pore size variability. Here, I prepared network realizations ranging from nearly homogeneous (*CV* = 0.05) to extremely heterogeneous (*CV* = 1.05). I also varied pore connectivity by randomly removing a fraction of the pipes and thus decreasing the coordination number *z* (mean number of connected pipes per nodes in the network). The coordination numbers considered here ranged from 3 to 6, corresponding to half and fully filled SC networks, respectively. The critical coordination number *z*_*c*_ at the percolation threshold is approximately equal to 1.5 in all three-dimensional networks^32^. For all combinations of *R*_*H*_, *CV* and *z*, multiple 12 × 12 × 12 and 16 × 16 × 16 network realizations were constructed. For the bimodal realizations, two populations of pipe with very narrow radii distributions (*CV* = 0.05) were considered. The set of large pipes had a hydraulic radius ten times greater than that of the small pipes (the pipe length was equal to ten times the hydraulic radius of the large pipes population). Five combinations of large and small hydraulic radii was used, i.e., 30/3, 10/1, 3/0.3, 1/0.1 and 0.3/0.03 10^−6^ m. In each realization, the individual pipes were randomly assigned one or the other distribution as specified by pre-selected number fractions of large and small pipes, *w*_L_ and *w*_S_ = 1 − *w*_L_. The pipe radii were then accordingly drawn. No pipes were removed, implying *z* = 6 in all bimodal realizations. However, the separate coordination numbers of large and small pipes, *z*_L_ and *z*_S_, varied between 0 and 6 depending on *w*_L_.

### Gas flow simulations

Simulating fluid flow through pipe networks consists in solving the Kirchoff equations, i.e., to impose local conservation of mass at each node *i* as ${\sum }_{a}{\dot{m}}_{i\alpha }$ = 0, where the subscript α refers to the nodes connected to *i* and the mass fluxes can be expressed using the appropriate one among equations 1–5 (note that, for the sake of simplicity, pure Poiseuille flow was assumed and entrance flow corrections were not included in the Kirchoff equations; the pipe length was generally chosen long enough to validate this assumption). One important difference of gas and incompressible liquid flow is that the laws of transport through individual bonds are non-linear in the case of gas flow, whereas they are linear for incompressible liquids. As a consequence of the non-linearity, the Kirchoff equations are much harder to solve for gas than liquid flow simulations. Limitations in computer power prevented me from utilizing the high-performance, parallelized, successive over-relaxation (SOR) iterative solver of Li *et al*.^17^. Instead, I implemented a simple, basic version of the relaxation method. The slowness of my iterative non-linear solver imposed strong restrictions on the number and size of the simulations that I could be run in a realistic time frame. In particular, I could not carry out the Bernabé *et al*.^30^ technique of using different types of networks (i.e., simple cubic, BCC and FCC) to test the generality of the results. Only simple cubic simulations were performed here (note that Li *et al*.^17^ obtained results from BCC and FCC simulations quite consistent with the simple cubic ones).

**Figure 2:**
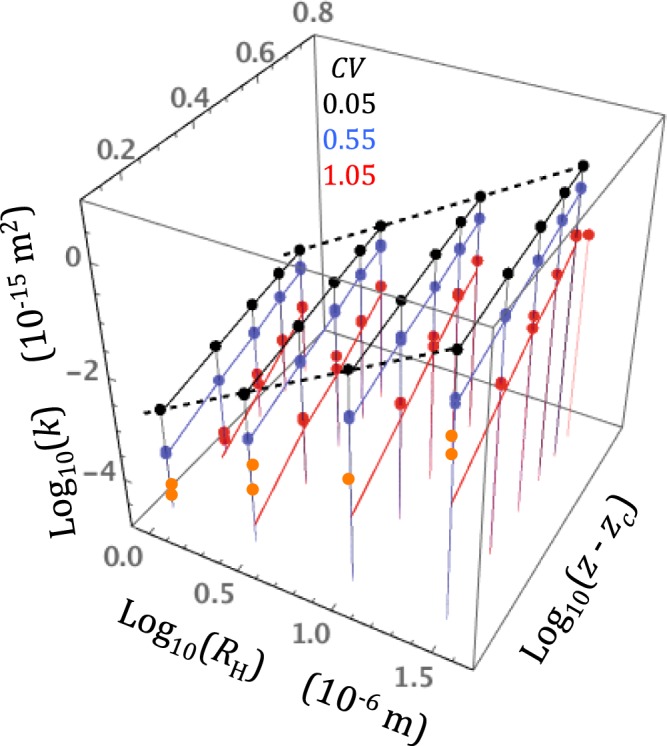
A three-dimensional plot of ensemble averaged values of *k* as functions of *R*_H_ and (*z* – *z*_c_) for three values of *CV* (0.05, black dots, 0.55, blue dots and 1.05 red and orange dots). Incompressible liquid flow was performed in the same conditions. The color-matching solid and dashed lines in constant *R*_H_ and constant (*z* − *z*_c_) planes help visualize the liquid permeability field *k*_*L*_ for comparison with *k*. For the lowest values of *CV*, *k* and *k*_*L*_ are in good agreement while *k* is clearly greater than *k*_*L*_ for *CV* = 1.05 (the data-points with *k*/*k*_*L*_ > 2 are indicated in orange).

The so-called permeameter boundary conditions were used (constant pressures applied to the entry and exit faces, no flow allowed through the sides). To test the accuracy of the solver I compared the intrinsic permeability simulated using a gas in the purely viscous regime (equation 1) and the permeability *k*_*L*_ of an incompressible liquid through identical network realizations (in liquid flow simulations, the linear Kirchoff equations were solved using the Krylov method). In principle, one would expect *k*_*L*_ and *k* to be equal, but I found that the solver yielded values of *k* greater than *k*_*L*_. The difference between *k* and *k*_*L*_ depended on the pore radius variability measure *CV* and, to a lesser extent, to the pore coordination number *z*. The ratio *k*/*k*_*L*_ ranged from 1 to 1.1 for nearly homogeneous networks (*CV* = 0.05), from about 1.1 to 1.4 for *CV* = 0.55 and reached much greater values (from 1.3 to as high as 20) for the most heterogeneous networks (*CV* = 1.05). The largest ratios occurred when the connectivity was low (*z* ≈ 3). These observations are illustrated in Fig. 2, which shows a three-dimensional plot of ensemble averaged values of *k* as functions of *R*_H_ and (*z* − *z*_c_), and, a schematic visualization of the liquid permeability field as a series of lines in constant *R*_H_ and constant (*z* − *z*_c_) planes. The *k*_*L*_ lines have the form *k*_*L*_ = *C R*_H_^2^ (*z* − *z*_c_)^β^ predicted by the Bernabé *et al*.^30^ model (the numerical values of *C* and β specifically associated with *CV* = 0.05, 0.55 and 1.05 are given in Bernabé *et al*.^30^).

Although the solver used here was accurate in nearly homogeneous networks, the accuracy could be deteriorating in networks with increasing heterogeneity, thus casting doubts about the validity of the *k*/*k*_*L*_ > 1 inequality. Alternatively, the increase of *k*/*k*_*L*_ with increasing heterogeneity may reflect a real gas flow property. This issue is difficult to resolve. Since the accuracy of the solver depends on the assumed initial pressure field (IPF), I tried several possible IPF’s, including the pressure field predicted by equation 1 for a perfectly homogeneous network (*R*_*i*_ = *R*_*H*_ for all pipes) or that produced by the liquid flow simulation. The diverse solutions obtained did not differ by more than 1 or 2 percent. I also tried to change the solver arrest condition in purely viscous gas flow simulations and found that the intrinsic permeability increased with tightening of the arrest condition. A tighter arrest condition presumably leads to improved accuracy, albeit at a great cost in CPU time. Thus, the observed increase of *k*/*k*_*L*_ with increasing heterogeneity is in fact more likely to be a valid result than not. In any event, to avoid including potentially spurious results in the subsequent analyses, I discarded the highly heterogeneous network realizations, for which *k*/*k*_*L*_ was larger than 2 (i.e., the orange data-points in Fig. 2).

Since the Knudsen number varies with gas pressure, gas flow for each network realization was simulated at 6 different mean macroscopic gas pressures $\bar{P}$ (namely, 10, 0.1, 0.01, 0.003, 0.001 and 0.0003 MPa). These variations in mean gas pressure combined with those of the hydraulic radius produced more than 6 orders of magnitude changes in Knudsen numbers, *K*_n_ = (μ/$\bar{P}$ *R*_*H*_) (π*R*_*g*_*T*/2)^1/2^. The maximum Knudsen numbers *K*_max_ achieved for each networks realization were 74, 22, 7.4, 2.2 and 0.74 corresponding to *R*_*H*_ = 0.3, 1, 3, 10 and 30 10^−6^ m, respectively. Varying $\bar{P}$ also allowed performing the Klinkenberg analysis for each network realization (i.e., examining the dependence of the gas permeability *k*_gas_ on the inverse gas pressure 1/$\bar{P}$).

Two different rules were used to set the macroscopic pressure difference Δ*P*, the small-gradient rule, Δ*P* = $\bar{P}$/10, and the large-gradient rule, Δ*P* = 2($\bar{P}$ − 0.0001). The large-gradient rule obviously produced much greater variations in local Knudsen numbers than the small-gradient rule, especially when the mean pressure $\bar{P}$ was high (it also yielded slightly higher *k*/*k*_*L*_ ratios). Only the small gradient-rule was used with the bimodal network realizations. All the simulations assumed that the saturating gas was nitrogen at room temperature. For simplicity, the compressibility factor *Z* was taken to be equal to 1 and full momentum accommodation was considered (σ = 1).

## Results

### Monomodal networks

**Figure 3:**
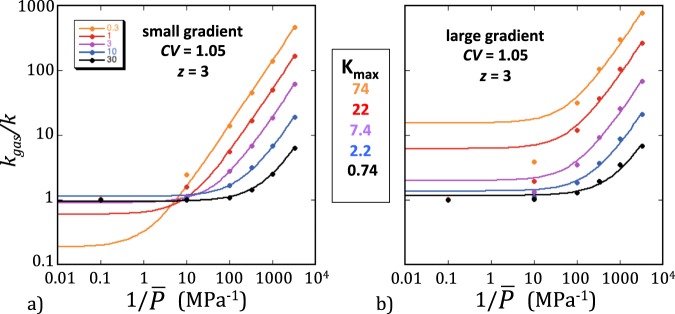
Examples of Klinkenberg plots of the simulated ratio *k*_*g*_/*k* as a function of the reciprocal mean gas pressures 1/$\bar{P}$ (*CV* = 1.05 and *z* = 3). The small-gradient rule was used in (**a**) and the large-gradient rule in (**b**). The values of *R*_H_ and *K*_max_ corresponding to each set of simulations are indicated in the insets in colors matching the data-points. The color-matching solid lines represent the best fitting linear functions, *k*_*g*_/*k* = A* + B*/$\bar{P}$. These lines show that the intercept A* was lower than 1 when the small gradient was used and greater than 1 otherwise.

**Figure 4:**
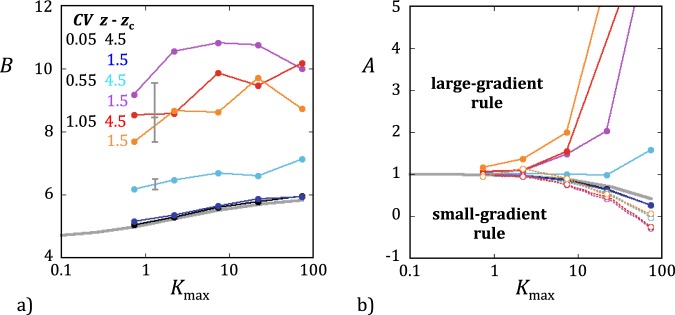
Values of (**a**) the slope *B* and (**b**) the intersect *A* of the best fitting linear function, *k*_*g*_/*k* = *A* + *B K*_n_, as functions *K*_max_. The values of *CV* and *z* − *z*_c_ used for each set of simulations are given in the inset in matching colors. The solid symbols and lines indicate that the large-gradient rule was used whereas the open symbols and dashed lines correspond to the small-gradient rule. The thick grey lines visualize *A* and *B* previously estimated for the function *F*.

For each network realization, values of *k* and *k*_*g*_ were calculated using equation 1 and equations 5 and 6, respectively. The simulated *k*_*g*_’s corresponding to different mean gas pressures $\bar{P}$ can conveniently be expressed in normalized form, *k*_*g*_/*k*, and displayed in Klinkenberg-type plots (Fig. 3). Linear functions, *k*_*g*_/*k* = A* + B*/$\bar{P}$, appeared to fit the simulated data very well in all cases, although a subtle underlying non-linearity was indicated by the dependence of A* and B* on the maximum Knudsen number reached for each *k*_*g*_/*k* curve. For comparison with *F*, the normalized gas permeability can be recast as a function of the Knudsen number

$${k}_{g}/k=A+B{K}_{{\rm{n}}},$$

where *A* and *B* depend on *K*_max_. The values of *A* and *B* in nearly homogeneous networks (*CV* = 0.05) were very close to those estimated from the function *F* and, furthermore, were not significantly affected by changes in connectivity (Fig. 4). This good agreement of *k*_*g*_/*k* and *F* in homogeneous networks was not preserved, however, for higher levels of heterogeneity. In network realizations with *CV* ≥ 0.55, *A* and *B* for *k*_*g*_/*k* increasingly diverged from their *F* counterparts when *CV* was increased and/or *z* decreased. But the most striking result was that the intersect *A* had completely different forms when the small- and large-gradient rules were used. With the small-gradient rule, the results were analogous to those shown in Fig. 1, namely, *A* was lower than 1 and decreased with increasing *K*_max_. In contrast, values of *A* larger than 1 and increasing with *K*_max_ were obtained when the large-gradient rule was applied (Fig. 4). An increase of *A* with *K*_max_ indicates a non-linear Klinkenberg curve with a downward curvature and implies an overestimation of *k* if the Klinkenberg extrapolation is used. The implication is thus that experimental Klinkenberg plots may yield incorrect underestimates or overestimates of the intrinsic permeability if the maximum Knudsen number investigated is larger than about 1 (a limit suggested by Fig. 4), depending on the heterogeneity level and the magnitude of the pressure gradient.

**Figure 5:**
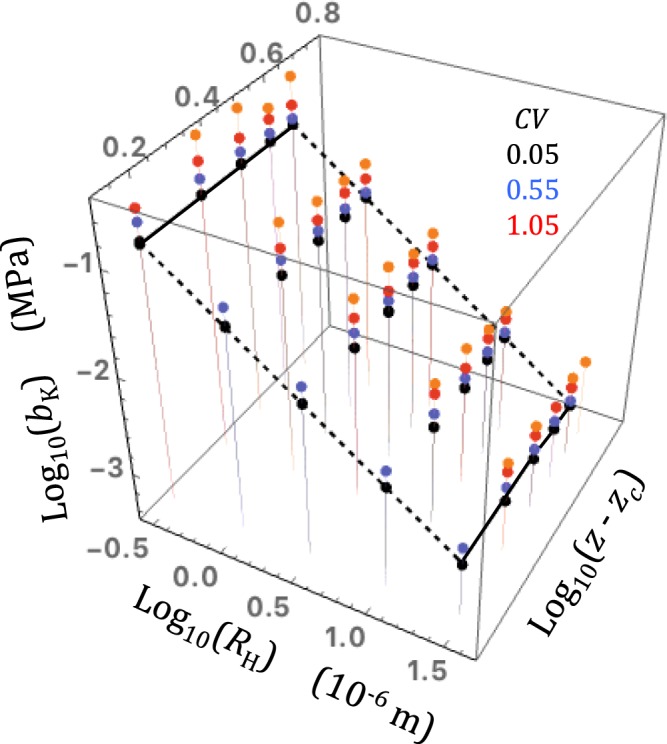
A three-dimensional plot of the Klinkenberg coefficient *b*_K_ for the various network realizations excluding the *k*_*g*_/*k* data obtained for *K*_n_ > 1 as a function of *R*_H_ and *z* − *z*_c_. The values of *CV* used in the simulations are given in matching colors (the orange dots represent the simulated *b*_K_ for *CV* = 1.05 using the large-gradient rule). The horizontal solid straight lines in constant-*R*_H_ planes demonstrate that *b*_K_ was essentially insensitive to connectivity changes. The inclined dashed lines in constant-(*z* − *z*_c_) planes show that *b*_K_ was linearly related to the inverse hydraulic radius.

Since the Klinkenberg analysis can be properly performed when *K*_max_ is lower than 1, I calculated the Klinkenberg coefficient *b*_K_ for the various network realizations excluding the *k*_*g*_/*k* data obtained for *K*_n_ > 1 (Fig. 5). In simulations using the small-gradient rule, *b*_K_ was approximately proportional to the inverse hydraulic radius, *b*_K_ ≈ C*/*R*_H_, with C* a relatively weak linear function of *CV* (specifically, C* = 0.034 + 0.2*CV*). Importantly, there was no discernable effect of connectivity (the data-points fell on nearly horizontal lines in constant *R*_H_ planes; Fig. 5). The large-gradient rule produced similar results with slightly larger and more variable values of *b*_K_ (Fig. 5).

### Bimodal networks

**Figure 6:**
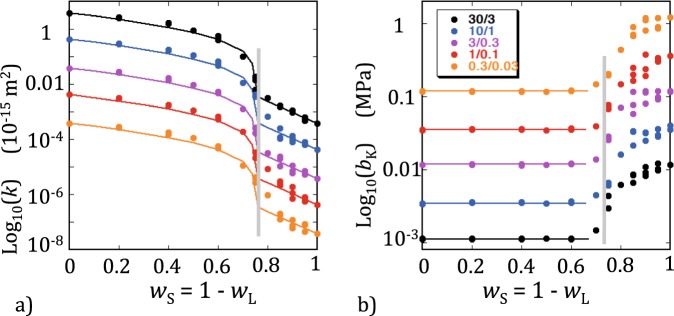
Results of the bimodal simulations. The variations with *w*_S_ of (**a**) *k* and (**b**) *b*_K_. The five combinations of large and small hydraulic radii used in the simulations are indicated in the inset in matching colors. The thick vertical grey line shows the location of the percolation threshold of the large pipe population. The discontinuous dashed lines in (**a**) show the good fit obtained with the modified Bernabé *et al*.^33^ model (see text and Appendix A for more details). The horizontal dashed lines in (**b**) demonstrate that *b*_K_ is nearly constant until the percolation threshold of the large pipe population is nearly reached.

The intrinsic permeability *k* generally decreased with increasing *w*_S_ and a sharp drop occurred when the set of large pipes became disconnected (i.e., for *w*_L_ ≈ 0.25 or *w*_S_ ≈ 0.75). The variations of *k* with *w*_S_, including the singularity observed above the large pipes percolation threshold (for *w*_L_ ≥ 0.25 or *w*_S_ ≤ 0.75; Fig. 6a), were similar for all hydraulic radii combinations and were well reproduced by a simple modification of the percolation-based binary mixing model of Bernabé *et al*.^33^. The Bernabé *et al*.^33^ model uses different mixing laws above and below the percolation threshold of the high-permeability phase, namely, the upper and lower Hashin-Shtrikman bounds for *w*_L_ > 0.25 and *w*_L_ < 0.25, respectively. A better fit was obtained here by replacing the lower Hashin-Shtrikman bound with the geometric average (see details in Appendix A).

The Klinkenberg coefficient *b*_K_ also displayed a typical percolation behavior. For each hydraulic radii combination, *b*_K_ remained nearly constant until *w*_S_ approached the percolation threshold, where a sudden jump occurred (Fig. 6b). Strikingly, the singularity was located below the large pipes percolation threshold (for *w*_L_ ≤ 0.25 or *w*_S_ ≥ 0.75; Fig. 6b) and not above as normally expected. Thus, the connectivity of the large pipes significantly affected *k* but had a negligible influence on *b*_K_ (this is similar to the lack of sensitivity of *b*_K_ to *z* − *z*_c_ in Fig. 5). Below the percolation threshold, the (disconnected) large pipes still strongly affected *b*_K_ while their effect on *k* was weak.

## Discussion

### Upscaling of the BK single pipe model

The relationship of *k*_*g*_/*k* and *K*_n_ obtained in the simulations, in effect, represents the result of upscaling the BK model from the scale of a single pore to that of a porous body containing many pores. One manifest result is that the simulated macroscopic behavior was not always similar to that predicted by the BK model. The simulated data revealed very weakly non-linear Klinkenberg curves, asymptotically becoming linear at high Knudsen numbers, as expected from the BK model. But both upward- and downward-bending of the Klinkenberg curves were observed whereas equations 5 and 6 only show upward curvature. Indeed, rare but clear instances of upward or downward bending of experimental Klinkenberg curves have indeed been observed^34–36^. However, the subtle non-linearity predicted by the simulations could often be obscured by experimental uncertainties.

The main factor producing weakly non-linear Klinkenberg curves in the simulations was the heterogeneity of the field of local Knudsen numbers, which itself resulted from the geometrical heterogeneity of the networks combined with the gas pressure difference imposed externally. The origin of the strong non-linearity occasionally observed in rocks is less clear. Rarefaction effects such as those predicted by the BK model must have played a role but were probably not the only cause since Knudsen numbers larger than 1 were rarely reached in these experiments (see Fig. 9 of Sinha *et al*.^34^ or Figures 14 and 15 of Wang *et al*.^36^). Other causal factors include deformability of the porous material, which may have a strong effect at high gas pressures (for example, in Fig. 7 of Wang *et al*.^36^ the increase of *k*_g_ as the inverse gas pressure approaches 0 was likely due to a decrease of the effective pressure; see also Letham and Bustin^37^), the non-ideal constitutive behavior of the gas^3^ and gas adsorption in porous media with a very small pore size^1^.

**Figure 7:**
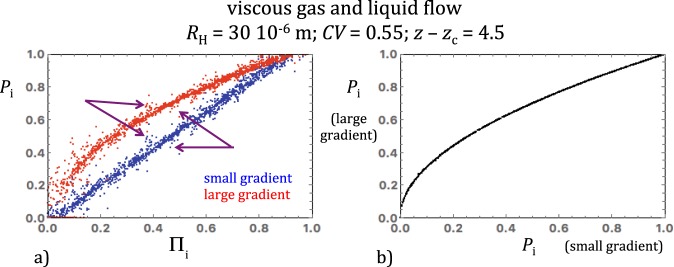
(**a**) Plot against each other of the normalized gas and liquid pressure fields, *P*_i_ and Π_i_, obtained for the same network realization (*R*_H_ = 30 10^−6^ m, *CV* = 0.55 and *z* − *z*_c_ = 4.5). The pressure fields *P*_i_ corresponding to the small- and large-gradient rules are drawn in blue and red, respectively. The arrows point to examples of the same large fluctuations that can be identified in both pressure fields. (**b**) Fluctuation-free cross-plot of the large- and small-gradient gas pressure fields for the same network realization.

### Difference of liquid and intrinsic gas permeability

Another important result is that the intrinsic permeability *k* estimated from purely viscous gas flow simulations was apparently larger than the liquid permeability *k*_L_. As discussed in section 2, there is a reasonable possibility that the enhanced efficiency of compressible flow with respect to incompressible flow was not a numerical artifact but resulted from genuine differences between the pressure fields produced in identical networks by flow of a compressible gas and an incompressible liquid. To illustrate this point, the normalized gas and liquid pressure fields, *P*_i_ and Π_i_, obtained for the same network realization (*R*_H_ = 30 10^−6^ m, *CV* = 0.55 and *z* − *z*_c_ = 4.5) were plotted against each other in Fig. 7a. Two versions of *P*_i_ corresponding to the small- and large-gradient rules are shown in the same diagram. The effect of gas compressibility is evident for the large-gradient dataset (quasi-quadratic *P*_i_ versus Π_i_ trend) while it is almost invisible in the small gradient case (quasi-linear trend). Most importantly for this discussion, the relationship between *P*_i_ and Π_i_ is blurred by strong fluctuations with respect to the overall trends. These fluctuations are irregular but not random as they resulted deterministically from the specific *R*_i_ field of this network realization. Under close examination, identical very strong fluctuations can easily be identified in the large- and small-gradient data-point clusters, implying that the geometrical heterogeneity of the network affected the two pressure fields in a nearly indistinguishable way. This view is supported by a cross-plot of the large- and small-gradient gas pressure fields, precisely outlining a fluctuation-free, compressibility-controlled relationship among the two pressure fields (Fig. 7b). Similar observations were made for network realizations with larger *CV* and/or smaller *z* – *z*_c_, although the level of numerical noise was higher than in Fig. 7.

Ratios of Klinkenberg-corrected gas permeability to liquid permeability, *k*/*k*_L_, significantly greater than 1 have been experimentally observed in crystalline rocks (Christian David and Jerome Wasserman, personal communication), in tight sandstones^38,39^ and other materials such as clay-bearing fault gouge^40^ or mortar^41^. The origin of the experimental *k* > *k*_L_ discrepancy remains unclear. The nature of the saturating liquid or gas appears to be very important. Permeability to water tends to be lower than permeability to non-polar liquids^41^, suggesting that water flow tests can be strongly affected by water-solid interactions (note that distilled water was observed to reduce permeability more than brine^38,40^). Gas flow experiments showed that permeability to nearly ideal gases such as helium is substantially higher than the permeability to non-ideal gases such as methane and that the difference increases with non-ideality^3,34^.

### Effect of the pressure gradient

**Figure 8:**
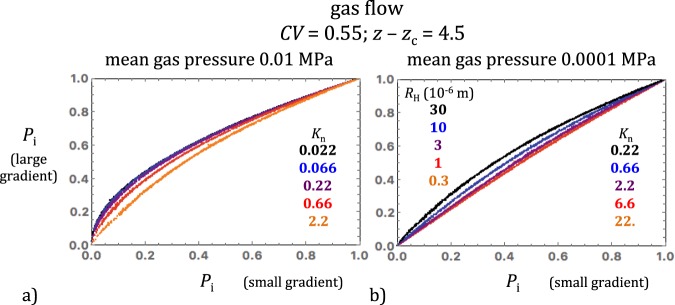
Cross-plots of the large- and small-gradient pressure fields simulated in the same realizations (*CV* = 0.55, *z* − *z*_c_ = 4.5) for two values of the mean gas pressure (**a**) 0.01 MPa and (**b**) 0.0001 MPa. The values of *R*_H_ and *K*_n_ used in these simulations are given in the insets in matching colors. The cross-plotted curves show very small fluctuations about the overall trends and thus demonstrate a regular transition with decreasing *K*_n_ from quadratic to nearly linear trends.

Besides the effects discussed above, the magnitude of the pressure gradient also affected the gas pressure fields simulated in conditions corresponding to moderate to high Knudsen numbers. Cross-plots of the large- and small-gradient pressure fields simulated in the same realizations are shown in Fig. 8 for $\bar{P}$ = 0.01 and 0.0001 MPa and various input parameters (*CV* = 0.55, *z* – *z*_c_ = 4.5 and *R*_H_ = 30, 10, 3, 1 and 0.3 10^−6^ m). The Knudsen numbers achieved in these simulations increased from 0.022 to 22 with decreasing *R*_H_ and $\bar{P}$. As in Fig. 7b, the cross-plotted curves show very small fluctuations about the well-delineated overall trends. The important observation is that these curves reveal a regular transition with decreasing *K*_n_ from quadratic to nearly linear trends (Fig. 8). Thus rarefaction and compressibility effects tend to balance each other in gas flow at large Knudsen numbers.

### Implications for the Klinkenberg analysis

The simulated gas flow at various gas pressures was compatible with the Klinkenberg analysis only when the maximum Knudsen number considered was lower than 1. Since the ultimate purpose of the Klinkenberg analysis is to determine the intrinsic permeability *k*, substantial errors in *k* can occur unless carefully selected experimental conditions are used: (a) the externally applied pressure difference across the sample should be minimized, (b) the highest mean gas pressure reached during the measurements should be much smaller than the confining pressure to reduce variations in effective pressure and the associated pore space deformations, (c) the lowest mean gas pressure should correspond to a Knudsen number no greater than 1, and finally, (d) substantially non-ideal gases should be avoided (e.g., methane, carbon dioxide).

**Figure 9:**
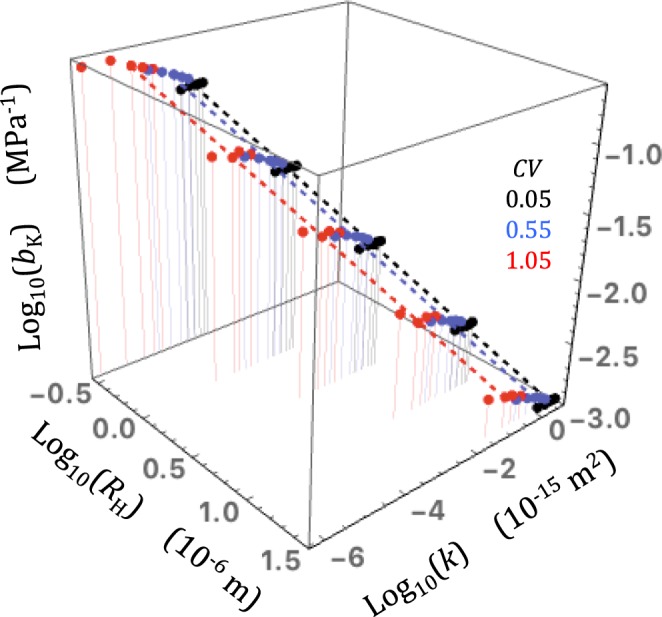
A three-dimensional plot of *k*, *b*_K_ and *R*_H_ for monomodal network realizations. The values of *CV* are indicated in the inset in matching colors. The color-matching dashed straight lines visualize the power laws, *k* ∝ *R*_H_^2^ and *b*_K_ ∝ 1/*R*_H_ approximately followed by the simulated *k* and *b*_K_.

The relationship between *k* and *b*_K_ is also an issue of great interest in rock physics. Experimental data suggest a power law relationship, *b*_K_ ∝ *k*^−*n*^. Values of the exponent *n* between 0.33 and 0.39 were obtained from large rock datasets^38,42^. However, these values can be considered uncertain owing to the very large scatter clouding the data. Wang *et al*.^36^ measured *k* and *b*_K_ in two gneiss samples subjected to increasing confining pressures. They found very well defined power laws with *n* = 0.20 and 0.53, demonstrating that the relationship between *k* and *b*_K_ is highly variable for individual rocks. Civan^22^ derived a model predicting a slightly different power law, *b*_K_ ∝ (*k*/ϕ)^−*n*^, where ϕ denotes porosity and *n* = 1/2 (Sampath and Keighin^39^ applied this model to their data and found *n* = 0.53). The values of *k* and *b*_K_ obtained here in monomodal simulations are represented in a three-dimensional diagram against each other and the hydraulic radius *R*_H_ of the network realizations (Fig. 9). The diagram shows that, as already mentioned in sections 3 and 4, *k* and *b*_K_ are very sensitive to the hydraulic radius and generally obey the power laws, *k* ∝ *R*_H_^2^ and *b*_K_ ∝ 1/*R*_H_ (the dotted lines shown in Fig. 9 for comparison were calculated such that their projections on the *k* − *R*_H_ and *b*_K_ − *R*_H_ planes obeyed these power laws). The intrinsic permeability is also significantly affected by pore size heterogeneity *CV* and pore connectivity *z* − *z*_c_ while the Klinkenberg coefficient is weakly affected by *CV* and almost totally insensitive to *z* − *z*_c_ (Li *et al*.^17^ obtained very similar results using the Javadpour pipe model). Combining these power laws, one easily finds *b*_K_ ∝ *k*^−1/2^. However exponents lower than ½ as observed in natural rocks^38,42^ can be obtained if subsets of the simulated data are appropriately selected. Reduction of *n* is easily achieved by introducing some sort of correlation of *k* to *z* − *z*_c_ and anti-correlation to *CV*, a reasonable assumption since highly permeable rocks, indeed, possess better pore connectivity and tend to show less pore size relative variability than those with very low permeability.

### Bimodal networks

The bimodal networks investigated here are not realistic representations of rocks containing micro-porosity (a more truthful image is given by dual porosity networks^43^). The results obtained are nevertheless quite instructive. Both the intrinsic permeability and Klinkenberg coefficient displayed typical percolation singularities when *w*_L_ crossed the percolation threshold of the large pipe population (Fig. 6), although the behaviors of *k* and *b*_K_ were very different. The intrinsic permeability simulated data are very well reproduced using two different binary mixing laws, the upper Hashin-Shtrikman bound above (i.e., to the left of the grey line in Fig. 6a) and geometric averaging below the percolation threshold (see Appendix A). In this model, the percolation singularity results from the critical power law describing the number fraction of large pipes that belong to the connected cluster (here, I used the classic value, 0.41, of the critical exponent). In the case of the Klinkenberg coefficient, however, the singularity is located below the percolation threshold (i.e., to the right of the grey line in Fig. 6b). Remarkably, *b*_K_ appears to be constant, independent of *w*_L_, above the percolation threshold. This peculiar behavior may be related to the insensitivity of *b*_K_ to *z* – *z*_c_ observed in monomodal simulations. This result suggests that, in dual porosity rocks, *b*_K_ may be insensitive to the presence of micro-porosity as long as the macro-pore population is connected.

## Conclusions

- The flow of an ideal gas through heterogeneous and imperfectly connected simple cubic networks of pipes was simulated numerically. The simulations included both rarefaction and compressibility effects. The permeability to gas in the purely viscous regime (*K*_n_ = 0) was found to be greater than the permeability to an incompressible liquid. This result suggests that a compressible fluid may flow through a heterogeneous porous media more efficiently than an incompressible one. Non-ideal constitutive laws may have an important additional effect.
- The functional dependence of gas flow on the macroscopic Knudsen number differed from that predicted by the BK single pipe model and was contingent on the pipe radius distribution, the pore connectivity and the magnitude of the externally applied pressure gradient. The implication in terms of the Klinkenberg analysis was that extrapolation of a Klinkenberg curve cannot be trusted to provide an accurate estimate of the intrinsic permeability unless the maximum Knudsen number investigated is lower than 1.
- The Klinkenberg analysis was applied to appropriate subsets of the simulated data (*K*_max_ < 1). The Klinkenberg coefficient *b*_K_ is almost completely insensitive to pore connectivity, only moderately affected by the width of the pipe radius distribution, and thus nearly proportional to the inverse hydraulic radius of the network.
- In the bimodal simulations, the intrinsic permeability *k* displayed a typical percolation behavior, with a singularity occurring immediately above the percolation threshold of the large pipe population. The Klinkenberg coefficient *b*_K_ showed an unusual behavior. It remained constant above the percolation threshold and developed a typical percolation singularity below it.

## Electronic supplementary material

Appendix A

## Data Availability

The data are available upon request at yvb@mit.edu.
