# Supplementary material for: Gaseous flow through heterogeneous, partially connected networks of pipes
Source: Sci Rep. 2018 Oct 8;8:14956. doi: 10.1038/s41598-018-33374-2 (PMC6175833; doi:10.1038/s41598-018-33374-2)
Supplement: Supplementary file 1 — Appendix A [file 41598_2018_33374_MOESM1_ESM.docx]

**Gaseous flow through heterogeneous, partially connected networks of pipes**

Yves Bernabé

Earth, Atmospheric and Planetary Sciences Department, Massachusetts Institute of Technology, Cambridge, Massachusetts, USA.

**Appendix A**

In bimodal networks, the sets of large and small pipes are called (L) and (S), respectively. The (L) and (S) populations are characterized by the hydraulic radii *R*_L_ and *R*_S_ (with *R*_L_ = 10 *R*_S_ as explained in section 2). For modeling purposes, each member of (L) and (S) is assigned a permeability value, *k*^(L)^ and *k*^(S)^, corresponding to a perfectly homogeneous network with a hydraulic radius equal to *R*_L_ and *R*_S_, respectively.

Above the percolation threshold of the (L) population (i.e., for *w*_L_ > *w*_c_ = 0.25 in SC networks), the medium is a mixture of two phases, (1) the portion of (L) forming the through-going connected cluster and (2) a mixture containing the (S) population and the unconnected portion of (L). The number fractions of the elements belonging to (1) and (2) are *w*_1_ = *w*_L_[(*w*_L_- *w*_c_)/(1- *w*_c_)]^0.41^ and *w*_2_ = 1 – *w*_1_, respectively. The number fraction of elements of (S) and (L) in the mixture (2) are *w*_S2_ = *w*_S_/*w*_2_ and *w*_L2_ = 1 – *w*_S2_. For *w*_L_ > *w*_c_, the permeability of the medium is given by the upper Ashin-Shtrikman bound:

$k=k^{\text{(L)}}\frac{w_{2}}{\frac{1}{k_{2}-k^{\text{(L)}}}+\frac{w_{1}}{3k^{\text{(L)}}}}$ (A1).

The permeability of the mixture (2), *k*_2_, is the geometric average of the (S) and (L) components of (2):

$k_{2}=k^{(S)^{w_{S2}}}k^{(L)^{w_{L2}}}$ (A2).

Below the percolation threshold (i.e., for *w*_L_ < *w*_c_), *k* is assumed equal to the geometric average of the (S) and (L) populations:

$k=k^{(S)^{w_{S}}}k^{(L)^{w_{L}}}$ (A3).

In the original Bernabé et al.^33^ model, the geometric averaging equations A2 and A3 were replaced by the lower Hashin-Shtrikman bound, but geometric averaging actually produced a better fit with the present simulated data.
